# Nanopore sequencing and hybrid assembly: unraveling the genomic landscape of dollar spot with enhanced annotation and drug resistance profiling

**DOI:** 10.3389/ffunb.2025.1621663

**Published:** 2025-07-17

**Authors:** Xiaojing Shi, Shu Zhao, John G. Gibbons, Geunhwa Jung

**Affiliations:** ^1^ Stockbridge School of Agriculture, University of Massachusetts, Amherst, MA, United States; ^2^ Department of Food Science, University of Massachusetts, Amherst, MA, United States

**Keywords:** genome assembly, comparative genomics, demethylation inhibitor, propiconazole resistance, clarireedia jacksonii

## Abstract

The increasing multi-drug resistance observed in the turfgrass pathogen *Clarireedia* spp. has emerged as a critical issue. Understanding the mechanisms underlying fungicide resistance is crucial to address this challenge. This study focuses on comparing a highly propiconazole-resistant isolate of *Clarireedia jacksonii*, HRI11, with a sensitive isolate, HRS10. Genomes were sequenced using the Oxford Nanopore MinION sequencing platform, and hybrid assembly was performed using this data and existing Pacific Biosciences long reads and Illumina short reads. HRI11 genome assembly represents the most contiguous and complete genome assembly reported for *Clarireedia* to date, spanning 43.6 MB with 12,831 predicted protein-coding genes across 51 scaffolds. In contrast, the HRS10 had an assembly size of 39.6 MB and encoded 12,161 putative proteins distributed over 100 scaffolds. While the two isolates share substantial sequence similarity and overall protein content, the fungicide resistance observed in HRI11 appears to arise primarily from genetic variants, particularly in genes encoding transcription factors, transporters, and fungicide target genes. These genetic variants establish a foundational resistance level against fungicides. Furthermore, induced resistance in HRI11 involves increased expression of proteins that facilitate fungicide efflux, thereby optimizing energy allocation during fungicide exposures. Together, these mechanisms-inherent genetic variation and adaptive transcriptional responses-contribute to the heightened resilience of HRI11 under fungicide treatment.

## Introduction

Dollar spot, caused by the ascomycete fungus *Clarireedia* spp. (formerly *Sclerotinia homoeocarpa*), is recognized as the most widespread and damaging disease affecting turfgrass (Burpee, 1997). Dollar spot leads to leaf damage and eventual death of turfgrass, significantly impacting their aesthetic value and playability. Turfgrass plays a vital economic and environmental role in modern society. In the United States alone, the turfgrass industry is valued at over $60 billion annually, supporting a wide range of sectors including landscaping, sod production, lawn care, and the maintenance of golf courses and athletic fields (Haydu et al., 2006). Beyond it economic significance, turfgrass enhances property values, supports recreational and tourism infrastructure, and contributes ecosystem services such as erosion control, water filtration, and urban heat mitigation (Braun et al., 2024). Given its broad utility and high maintenance requirements, ensuring the health and sustainability of turfgrass systems is both an economic necessity and an environmental priority.

Conventional management of dollar spot typically involves multiple applications of fungicides throughout the growing season. Left untreated, dollar spot can escalate to severe levels, reaching up to 90% disease severity (Allen et al., 2005). *Clarireedia* spp. has a broad host range, infecting all commonly cultivated cool-season and warm-season turfgrasses, making it one of the most economically significant diseases in the turfgrass industry (Sapkota et al., 2022). To date, the six recognized species within the genus *Clarireedia* - *C. homoeocarpa, C. bennettii, C. jacksonii, C. monteithiana, C. paspali*, (Sapkota et al., 2022) and *C. hainanense* (Zhang et al., 2022) - vary in geographic distribution and host preference. In North America, *C. jacksonii* is the dominant pathogen on cool-season turfgrass, while *C. monteithiana* primarily infects warm-season turfgrass.

Since the late 1960s, the development of site-specific fungicides has increasingly led to fungicide resistance in dollar spot (Brent and Hollomon, 2007). Moreover, the global rise in antifungal resistance over the past three decades poses a significant threat to human health and food supply (Fisher et al., 2018). Dollar spot has developed tolerance or resistance to various fungicide classes such as benzimidazole, dicarboximide, demethylation inhibitor (DMI), and succinate dehydrogenase inhibitor (SDHI), as well as multidrug resistance, persisting for over 50 years due to repeated fungicide applications (Green et al., 2016; Hulvey et al., 2012; Sang et al., 2018). Despite the long history of antifungal resistance in dollar spot, the genetic mechanisms specifically underlying DMI resistance remains poorly characterized.

Hulvey et al. (2012) identified *Sh*atrD as an ATP-binding cassette (ABC) efflux transporter responsible for DMI fungicide resistance of *C. jacksonii* under field conditions. Ma & Tredway (2013) demonstrated that induced overexpression of cytochrome P450 monooxygenase *Sh*CYP51 contributes to DMI resistance in the same species. Additionally, Sang et al. (2015) reported that the ABC efflux transporter *Sh*PDR1 mediates resistance to DMIs, as well as tolerance to dicarboximides and SDHIs in *C. jacksonii*. They also found that polymorphisms in the histidine kinase gene *Sh*os1 and overexpression of *Sh*PDR1 are responsible for dicarboximide resistance (Sang et al., 2017). Furthermore, Sang et al. (2018) showed that a gain-of-function mutation in the transcription factor *Sh*XDR1 leads to multi-drug resistance in *C. jacksonii* by inducing overexpression of CYPs and ABC efflux transporters. Finally, mutations in the target genes *Sdh*B or *Sdh*C were implicated in SDHI resistance in *Clarireedia* spp (Lee et al., 2021; Popko et al., 2018).

Oxford Nanopore MinION sequencing has significantly advanced multiple areas of molecular biology by providing real-time, portable, and cost-effective sequencing of DNA and RNA. Its long-read capabilities have broadened the scope of research across genome assembly, transcriptomics, epigenetics, and metagenomics. By directly reading tens of kilobases from single DNA molecules without the need for synthesis or amplification, the MinION platform simplifies *de novo* genome assembly and enhances existing reference genomes. These advances make it an invaluable tool in fields such as microbiology, epidemiology, and taxonomy (Kono and Arakawa, 2019). Complementing this technology, RNA-seq has transformed large-scale gene expression studies, providing comprehensive transcriptome data that not only enables gene identification but also improves genome annotation (Marioni et al., 2008). When integrated with annotated genomes, RNA-seq facilitates detailed transcriptome mapping and functional gene discovery. For example, Huangwei et al. (2023) used transcriptional profiling to investigate multi-drug resistance (MDR) mechanisms in *C. jacksonii*, underscoring the value of such integrative approaches.

Despite advancements in sequencing technologies, obtaining complete and high-quality genome assemblies for *Clarireedia* spp. remains a significant challenge. To date, genome sequences for twenty *Clarireedia* spp. isolates exist, but most are in draft form and highly fragmented, typically comprising over 1,600 scaffolds. Recently, Bahri et al. (2023) reported a genome assembly and annotation for the *C. aff. paspali* isolate H3, which improved continuity to 109 contigs; however, the assembly is not currently available in public repositories. Even with this progress, gene searches in the National Center for Biotechnology Information (NCBI) database are still severely hindered by the fragmented and incomplete nature of existing genome data. Currently, only 411 proteins (including 155 identical protein groups) are available on NCBI. This is significantly lower than expected for a filamentous fungus with a 40 Mb genome size, which typically encodes ~10,000 proteins., underscoring the urgent need for more complete and well-annotated genome resources.

Understanding the genetic mechanisms underlying fungicide resistance is essential for improving disease management in turfgrass systems. In *C. jacksonii*, resistance to demethylation inhibitor (DMI) fungicides such as propiconazole poses a significant challenge to effective and sustainable control strategies. However, existing genome assemblies and annotations for this pathogen remain highly fragmented and insufficiently curated, hindering in-depth functional and comparative genomic studies. To address these limitations, we present improved genome assemblies, functional annotations, and a comparative genomic analysis of two *C. jacksonii* isolates exhibiting differential resistance to propiconazole. Our study enhances the genome toolkit available for *Clarireedia* research and contributes to a deeper understanding of the molecular basis of DMI resistance in this important turfgrass pathogen.

## Materials and methods

### Sample isolates

Two isolates of *C. jacksonii*, HRS10 and HRI11, which previously had draft *de novo* genome assemblies (Green et al., 2016), underwent additional sequencing using the Oxford Nanopore platform, followed by *de novo* assembly and annotation. HRS10 exhibits sensitivity to multiple fungicide classes, whereas HRI11 shows multidrug resistance to demethylation inhibitor fungicides as well as other fungicide classes including plant growth regulators (Green et al., 2016; Hulvey et al., 2012). The genome reads generated by Green et al. (2016) for these isolates are accessible on GenBank under accession numbers LNGN00000000 for HRS10 and LNKV00000000 for HRI11.

### Genomic DNA extraction

Mycelia stored previously were incubated in potato dextrose broth (PDB) (Difco Laboratories, Detroit, MI) in a 50ml Falcon tube for 4 days, followed by sub-culturing in a 250 ml flask with PDB for an additional 4 days. Each isolate’s mycelia were then divided into six 2 ml centrifuge tubes. After brief centrifugation, any remaining media was discarded. Genomic DNA was extracted using a modified cetyl trimethyl ammonium bromide (CTAB) method (Cubero et al., 1999), with an incubation temperature of 65°C maintained for 30 minutes. Following the initial washing step with chloroform: isoamyl alcohol (24:1 v/v), every two upper aqueous phases were combined into a single tube to concentrate the final product. To ensure gDNA purity, precipitation was achieved using 100% ethanol. DNA concentration was quantified using the Qubit dsDNA HS Assay (ThermoFisher SCIENTIFIC), and sample purity was assessed via gel electrophoresis and by measuring the OD_260_/OD_280_ ratio with a Nanodrop.

### Oxford Nanopore MinION library preparation and sequencing

Genomic DNA was prepared for sequencing using the ONT SQK–RAD004 genomic rapid sequencing kit (Oxford, UK). Genomic DNA was fragmented using ONT Fragmentation Mix. To achieve this, 500ng of genomic DNA was gently mixed with 2.5 µL Fragmentation Mix in a PCR tube, then incubated at 30°C for 1 minute followed by 80°C for 1 minute. The tubes were subsequently cooled on ice. Next, the Rapid Adapter was ligated to the DNA ends by gently mixing 1 µL of Rapid Adapter with the DNA ends and incubated for 5 minutes at room temperature. The DNA library was then kept on ice until loading into the flow cell.

For flow cell preparation and library loading, reagents for priming and sequencing were thawed and mixed at room temperature and immediately transferred to ice upon thawing completion. The flow cell priming mix was prepared by pouring 30 µL of Flush Tether into the Flush Buffer tube, gently mixed by pipetting, and 800μl of the mix loaded into the flow cell through the priming port. After a 5-minute wait, the SpotON sample port cover was lifted and left open, and 200μl of priming mix was added through the priming port.

In a new tube, 34μl sequencing buffer, 25.5 µL of loading beads, 11μl of DNA library, and 4.5μl of nuclease-free water were combined. The mixture was added dropwise to the flow cell through the SpotON port. After adding the sample, the SpotON port cover was replaced, and the priming port was closed. The MinION lid was then replaced, and sequencing was initiated. The sequencing and base-calling processes were managed using the MinKNOW software. Base-called reads were subsequently downloaded for further analysis.

### De-novo genome assembly and quality assessment

The Illumina HiSeq2000 and Pacbio CLR reads for HRS10 and HRI11 were retrieved from a public database (Green et al., 2016). Raw Nanopore ONT reads were generated after 12 hours of sequencing. Quality control of the raw genomic reads was performed using FastQC v0.11.5 (Andrews et al., 2015). Sequence duplication for Illumina reads were collapsed into a single copy using Tally-15-065 (Richard et al., 2016). Trim-galore v0.4.2 (Krueger, 2015) was utilized to detect and trim adapter contamination as well as low-quality bases. The Canu v1.8 pipeline (Danecek et al., 2011) with default parameters was employed to generate corrected sequence consensus and assemble Nanopore and PacBio reads.

The assembled contigs underwent polishing using Illumina paired-end reads mapped with bwa v0.7.17 (Cingolani et al., 2012) and Pilon v1.23 (Emms and Kelly, 2019). Redundans v0.14a (Nattestad and Schatz, 2016) was applied to reduce heterozygous regions by identifying and selectively removing alternative heterozygous contigs. Genome statistics were generated using QUAST v5.0.2 (Dobin et al., 2013). The completeness of the genome assemblies was assessed using BUSCO v4.0.4 (Simão et al., 2015),which compares the assemblies to universal single-copy orthologs.

### Genome annotation

The genomes of the two *C. jacksonii* assemblies were annotated using the Funannotate pipeline v1.8.15 (Palmer and Stajich 2020). Genome repetitive elements were soft-masked using RepeatMasker v4.1.6 (Smit et al., 2015) and RepeatModeler 2.0.5 (Smit and Hubley, 2015) with default parameters. *Ab initio* gene models were predicted using two protein predictors: AUGUSTUS 3.5.0 (Stanke et al., 2006) and GeneMark-ES (Borodovsky and Lomsadze, 2011). Due to licensing restrictions, GeneMark-ES was run separately, and its predictions were manually integrated. For AUGUSTUS, Funannotate’s fungal training set and default settings were applied.

Transcript gene models were predicted by PASA 2.5.3 (Haas et al., 2008) using *de novo* transcript assemblies generated by Trinity 2.8.5 (Grabherr et al., 2011)under default parameters. Protein-coding gene models were predicted by protein alignment to the genome using Diamond 2.1.8 (Buchfink et al., 2014) and polished by Exonerate 2.4.0 (Slater and Birney, 2005). EVidenceModeler 1.1.1 (Haas et al., 2008) was employed to build consensus gene models, filtering by length, spanning gaps, and considering transposable elements from both *ab initio* and evidence-based gene models with default weighting. Functional annotation of the gene models was performed using sequence similarity blast (Altschul et al., 1990) against several databases, including Pfam (v36.0) (Mistry et al., 2021), UniProt DB version 2023_05 (Bateman, et al., 2023), InterProScan5 (v97.0) (Jones et al., 2014), BUSCO (v 5.6.0) (Simão et al., 2015), EggNOG (v6.0) (Hernández-Plaza et al., 2023), MEROPS (v12.0) (Zhang et al., 2018), and Carbohydrate-Active enZYmes (CAZy) Database (Cantarel et al., 2008). Protein-coding genes were additionally annotated by their protein domains using InterProScan5. The Phobius (Käll et al., 2004) webserver predicted transmembrane topology and signal peptides. Secondary metabolite gene clusters were predicted using the antiSMASH v7 webserver (Blin et al., 2023). Gene Ontology (GO)terms (Kanehisa et al., 2016), eukaryotic orthologous groups, and Kyoto Encyclopedia of Genes and Genomes (KEGG) metabolic pathways (Yu et al., 2012) were used for gene classification. Lastly, the genome’s tRNA and anticodons were predicted using tRNAscan-SE (v2.0.12) (Chan et al., 2021).

### Whole genome comparison

Several analytical pipelines were employed to compare the structural differences between the two isolates. Whole-genome alignment and visualization of insertions/deletions in aligned regions were conducted using MUCmer from the MUMmer sequence alignment v4.0.0 package (Marçais et al., 2018). To assess structural variations (SVs), copy number variations (CNVs), and single nucleotide polymorphisms (SNPs), HRI11 reads were aligned to the HRS10 genome using Bwa v0.7.17 (Li and Durbin, 2010). Samtools v1.18 facilitated conversion to various file formats for subsequent variant discovery analysis (Li et al., 2009). All tools were run with default parameters unless stated otherwise.

Structural variations were detected using Sniffles2 (Smolka et al., 2024) and DELLY (Rausch et al., 2012), with annotation provided by SURVIVOR_ant v0.0.1 (Sedlazeck et al., 2017). CNVs discovery was complemented by CNVpytor (Panda et al., 2024) and the Control-freeC pipeline (Boeva et al., 2012). SNPs were identified using FreeBayes v1.3.8 (Garrison and Marth, 2012) and filtered using VCFtools v0.1.16 (Danecek et al., 2011) to remove indels, low coverage and/or quality sites. Filtered SNPs were analyzed and annotated by Snpeff v4.3 (Cingolani et al., 2012) to predict the coding effects of genetic variations. OrthoFinder (Emms and Kelly, 2019) provided comparative genomics statistics and phylogenetic orthology inference. Functional comparison was facilitated by the Funannotate pipeline. All tools were run with default parameters unless stated otherwise.

### Resistome analysis

The resistome (Wright, 2007) refers to the full collection of genes in an organism that confer resistance to antimicrobial agents, a concept originally developed in bacterial systems and later adapted to fungi for identifying antifungal resistance determinants. In our study, we employed two fungal resistome databases, MARDy (Mycology Antifungal Resistance Database) (Nash et al., 2018) and AFRbase (Antifungal Resistance Database) (Jain et al., 2023), to compare resistance genes in our isolates.

### Transcriptome analysis

To investigate the molecular mechanisms underlying DMI resistance, we performed transcriptomic profiling of two *C. jacksonii* isolates, HRS10 and HRI11 under both untreated and propiconazole-treated conditions (0.1 µg ml^-1^ for 1 hour). For each isolate and condition, two independent biological replicates were used, yielding a total of eight RNA-seq libraries. Detailed methods for RNA extraction, cDNA library preparation, and sequencing are described in a previous publication (Sang et al., 2018). Each library generated over 75 million paired-end reads using the Illumina HiSeq 2000 platform.

Initial quality control of raw RNA-seq reads was conducted using FastQC v0.11.5 (Andrews et al., 2015), and quality and adapter trimming was performed using Trim-galore v0.6.10 (Krueger, 2015) with settings identical to those used for genomic reads. Given our focus on resistance mechanisms, all RNA-seq reads were aligned to newly assembled reference genome HRI11 using the STAR 2.7.11b (Dobin et al., 2013), and gene counts were quantified using HTSeq 2.0 (Putri et al., 2022). DESeq2 (Love et al., 2014) was employed for count normalization and detection of differentially expressed genes (DEGs) between two *C. jacksonii* isolates before and after propiconazole treatment. A gene is considered significantly differentially expressed if its adjusted *P*-value was < 0.05 as calculated by DESeq2.

Annotation data packages and databases for *C. jacksonii* were constructed using AnnotationForge with SQLite. GO enrichment and KEGG pathway enrichment analyses for DEGs were performed using enrichGO and enrichKEGG functions from the clusterProfiler v3.16.0 R package (Yu et al., 2012). These enrichment analyses were performed based on the custom annotation package created for this study.

## Results

### Hybrid assembly and gene prediction

Genome assemblies for HRS10 and HRI11 were produced using a hybrid sequencing approach, combing approximately 8 million previously published reads (200x coverage) from Illumina and PacBio platforms with newly obtained long-read data from the Oxford Nanopore MinION (**Table 1**). The final assembly size for HRS10 was 39.6 Mb, comprising 100 scaffolds and an N50 of 1.39 Mb. In comparison, HRI11 yielded a larger cumulative assembly of 43.6 Mb across 51 scaffolds with an N50 of 1.57 Mb. Both assemblies showed significant improvements in contiguity and quality, with over 99.1% of assembled sequences incorporated into scaffolds exceeding 50 kb in length. Assembly completeness was evaluated using Benchmarking Universal Single-Copy Ortholog (BUSCO), revealing that 98% of single copy orthologs were present in both genomes, indicating high assembly integrity. Genome annotations using Funannotate identified 12,161 and 12,831 protein-coding genes in HRS10 and HRI11, respectively. Annotation datasets with a BUSCO transcriptome completeness score of 98% were obtained, indicating that the vast majority of conserved fungal genes were successfully identified and annotated. The high score reflects the high quality and completeness of the genome annotation. Additionally, 131 transfer RNA (tRNA) comprising 22 anticodons were predicted in HRS10, while HRI11 featured 130 tRNAs.

**Table 1:** Comparing of previously published Illumina and PacBio genomes with the current hybrid assemblies of two *C. jacksonii* isolates - the multi-drug resistant HRI11 and sensitive HRS10.

Assembly features	Illumina and pacBio		Hybrid assembly	
	HRS10	HRI11	HRS10	HRI11
Number of scaffolds	231	257	100	51
N50 scaffold length (Mb)	0.6	0.7	1.4	1.6
Longest scaffold (Mb)	1.7	1.9	3.2	4.1
Assembly length (Mb)	42.3	43.4	39.6	43.6
Repeat content of assembly (%)	3.01	3.12	9.07	11.63
Number of predicted proteins	12,216	12,912	12,161	12,831
GC content of genes (%)	43.35	43.83	43.57	41.72
Integrity of assembly	N/A	N/A	98.6%	98.5%
Integrity of transcriptome	N/A	N/A	98.1%	98.%

Assembly features are shown for each method and isolate. The integrity of the assembly and transcriptome refers to the predicted completeness based on benchmarking universal single-copy orthologs using BUSCO.

Compared to previous assemblies based solely on Illumina and PacBio reads, the hybrid assemblies showed substantial improvements, including fewer and smaller assembly gaps, larger contigs, and a 2-fold increase in both N_50_ values and the longest scaffold lengths. Improved repeat filtering also reduced repetitive content, minimizing the potential for false-positive results in subsequent analyses.

### Gene content comparison

**Figure 1:**
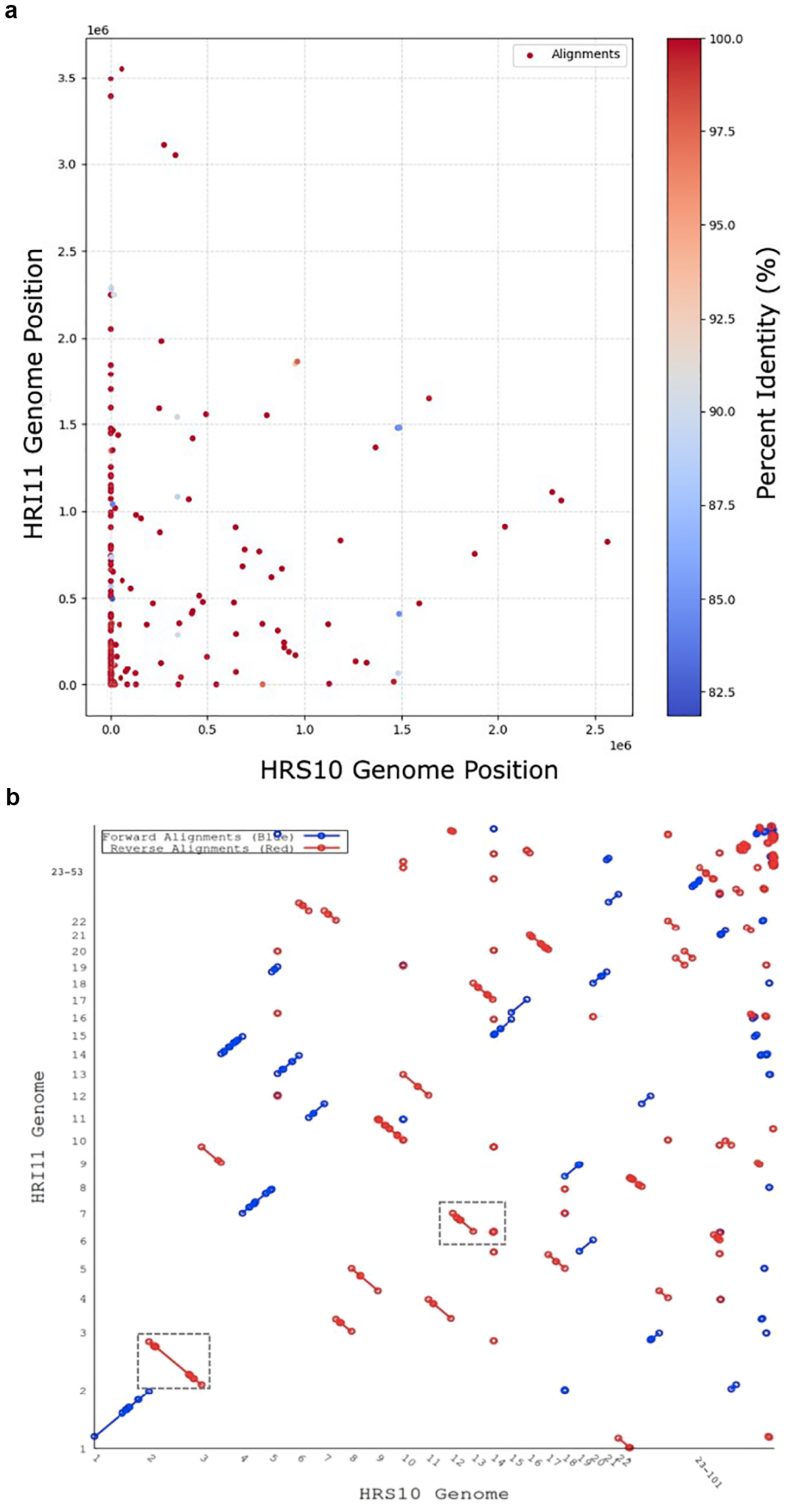
Genomic alignments between the multi-drug resistant isolate HRI11 and the sensitive isolate HRS10. **(a)** The x-axis represents the genomic position of HRS10, while the y-axis corresponds to the genomic position of HRI11. Each point represents an alignment, with the color scale indicating the percent identity of the alignments, ranging from 82.5% to 100%. Redder points signify higher sequence identity, whereas blue points indicate relatively lower sequence identity. **(b)** The x-axis represents the scaffolds of HRS10, while the y-axis represents the scaffolds of HRI11. Forward alignments are depicted in blue, while reverse alignments are shown in red. The boxed regions highlight inversions identified in scaffolds 2 and 7 of the HRI11 isolate.

Repetitive sequences, which often are a significant challenge in eukaryotic genome assemblies, complicating both assembly accuracy and functional prediction. To enhance genome quality, approximately 10% of these repetitive sequences were masked using the RepeatMasker program in both assemblies. As shown in **Figure 1**, the core genome shared between the two isolates consists of 10,797 orthogroups, representing 94.8% of all predicted genes. In addition to the core set, HRS10 and HRI11 possess 643 and 653 strain-specific genes, respectively (**Supplementary Table S1**). The majority of these unique genes encode hypothetical proteins with unknown functions, underscoring potential isolate-specific adaptions or functional divergence.

The observed differences in genome size and GC content may reflect natural intra-species variation, isolate-specific genomic features, or potential sequencing and assembly artifacts. Nonetheless, phylogenetic analysis strong evidence that that both isolates belong to the same species, *C. jacksonii*, supporting their suitability for comparative genomic and transcriptomic analyses.

### Genome structural comparison

Genome structural comparisons were performed using dedicated software to identify structural variation, copy number variation, and single nucleotide variation. Structural variations (SVs), which involve alterations in large segments of DNA, are significant drivers of genomic diversity. To analyze SVs between isolates HRS10 and HRI11, we employed DELLY and Sniffles2 pipelines with PacBio long reads. These pipelines revealed similar structural changes. Notably, HRI11 showed inversions on scaffolds 2 and 7 (**Figure 1b**), where these regions contained nine gene inversions. These genes include nucleoside triphosphate hydrolase (NTPases), two alcohol dehydrogenases, a member of the tetratricopeptide-like helical domain superfamily, and five hypothetical proteins. Insertion events affected two genes, causing a frameshift in the multidrug resistance gene *MDR1_3*. Moreover, the *NUD1* gene (leucine-rich repeat protein) had a breakpoint in HRI11. In addition, HRI11 exhibited deletions in two genes, *FSH3* (serine hydrolase) and *FUN_011878* (hypothetical protein), and *FUN_011921* (hypothetical protein) was duplicated.

CNVs between the two isolates, HRI11 and HRS10, were identified using both the CNVpytor and Control-FreeC pipelines, with cross-referencing to minimize false positives. HRI11 exhibited increased copy numbers for 31 genes and decreased copy numbers for 29 genes compared to HRS10 (**Supplementary Table S2**). Of the genes with increased copy numbers in HRI11, more than half are tRNA genes, including multiple copies of tRNA-Lys, tRNA-Gly, tRNA-Arg, and others. Additionally, 12 mRNA-encoding genes showed heightened copy numbers, with annotations including 10 hypothetical proteins, the global transcription regulator *ge1_2* (*SGE1*), and NADH:ubiquinone oxidoreductase subunit 5 (*nad5*). In contrast, all genes with decreased copy numbers in HRI11 are protein coding genes, including two ATP-dependent RNA helicases, *DED1* and *CHL1*.

**Figure 2:**
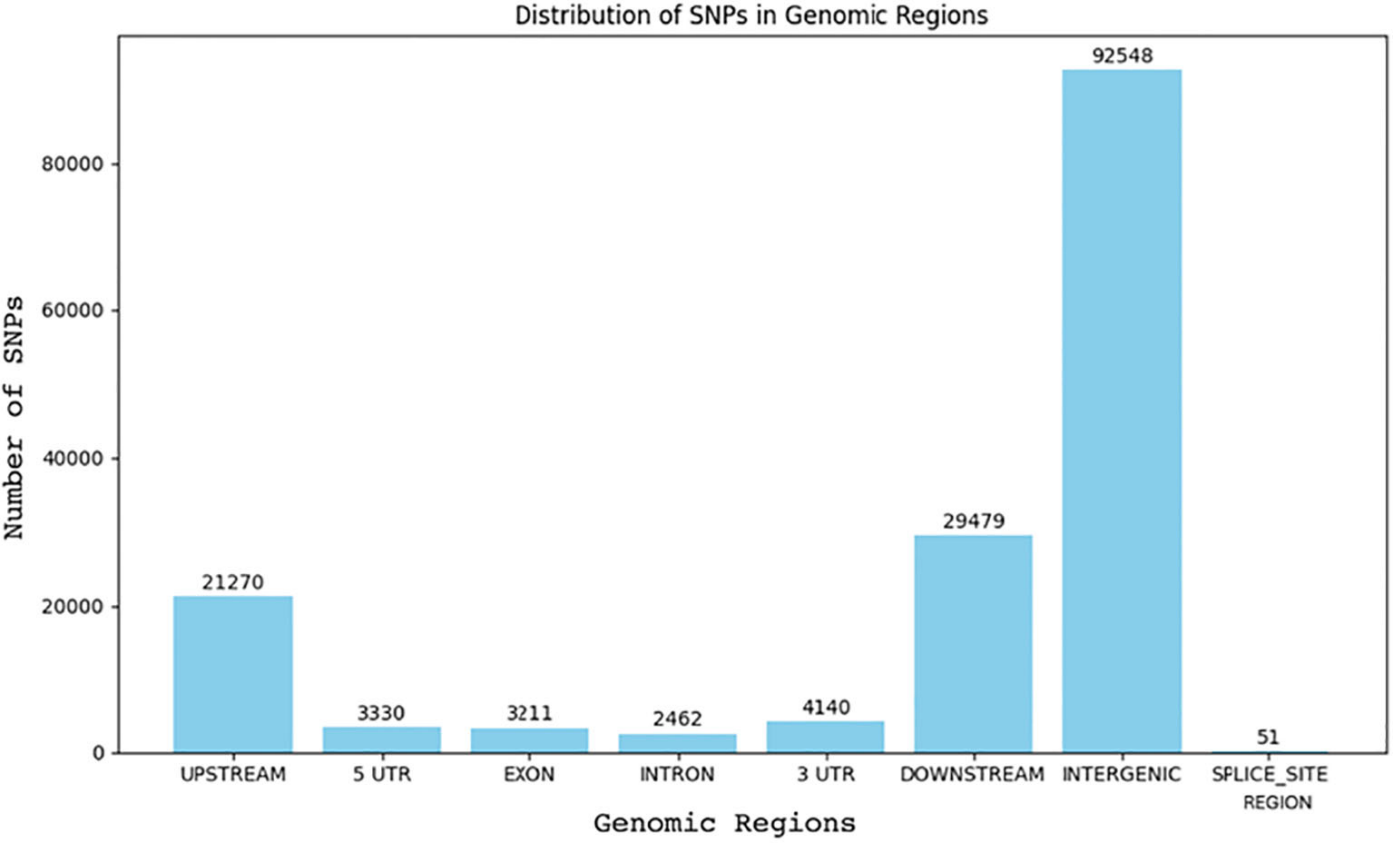
Distribution of SNPs across genomic regions in two *C. jacksonii* isolates: the multi-drug resistant HRI11 and the sensitive HRS10.

We identified 96,326 single nucleotide polymorphisms (SNPs) in the HRI11 isolate using FreeBayes, and these were analyzed for their effects using SnpEff. The majority of SNPs were located in intergenic regions (62.7%), followed by upstream (14.4%) and downstream (20.0%) regions (**Figure 2**). Less than 3% of the SNPs (3,215) were found within coding regions. Among these coding SNPs, approximately half (1,620) were synonymous, while the rest comprised 1,557 non-synonymous and 38 nonsense mutations. Analysis with Bcftools corroborated these findings, helping to refine the initial results. We identified 33 non-synonymous SNPs that overlapped between FreeBayes and Bcftools, which could have significant impacts on gene function. These SNPs predominantly caused start codon losses and premature stop codon mutations. While many affected genes are annotated as hypothetical proteins, their roles often relate to cellular metabolism and signaling pathways. Notably, a SNP was also found in the global transcription regulator *SGE1* within the HRI11 strain, underscoring its potential importance in observed genetic variations.

### RNA-Seq analysis

**Figure 3:**
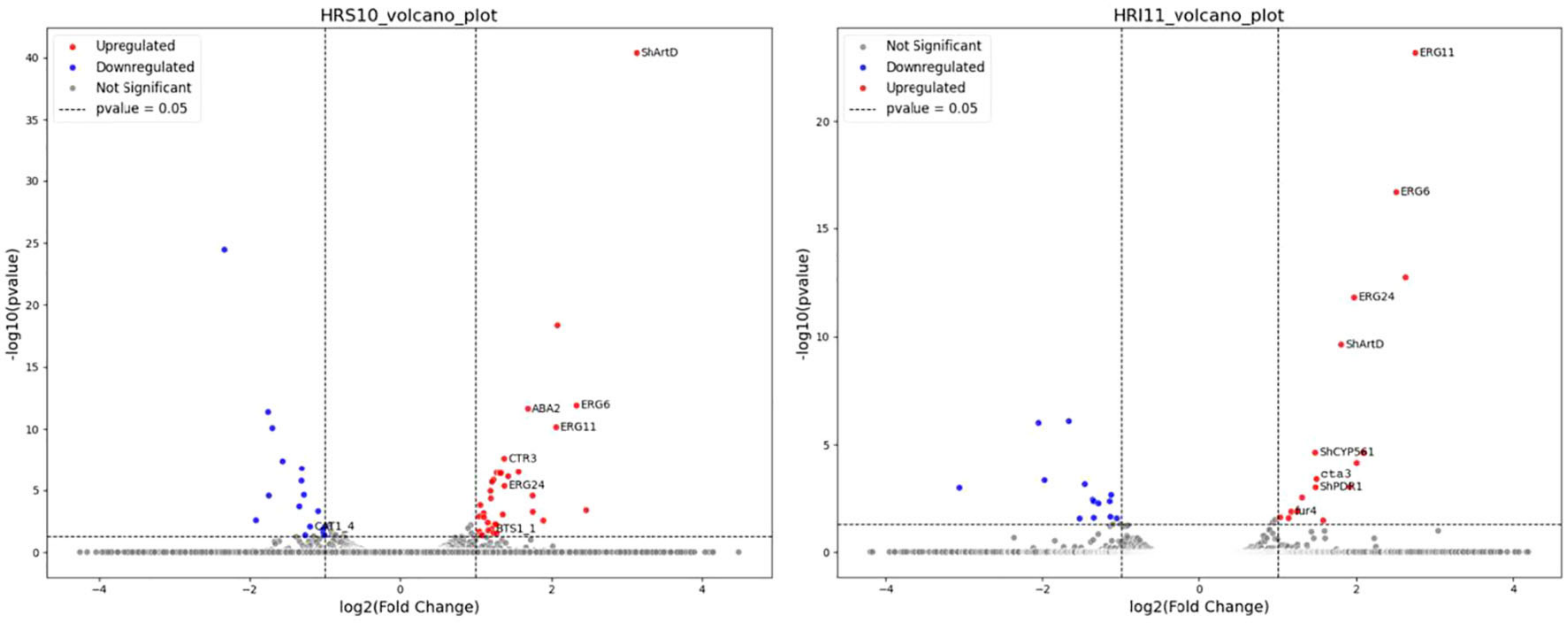
Volcano plot showing differentially expressed genes (DEGs) in response to azole, propiconazole treatment in the multidrug-sensitive HRS10 isolate (left) and the resistant HRI11 isolate (right).

RNA-seq analysis was conducted on two *C. jacksonii* isolates before and after propiconazole treatment. The analysis mapped RNA-seq reads to 96.1% of HRI11 gene models and 96.9% of HRS10 predicted genes. Genes showing significant differential expression were identified based on criteria of log2fold change > 1 and adjusted *P*-value < 0.05, calculated using DESeq2. Following propiconazole exposure, 37 genes in isolate HRS10 were upregulated, while 17 genes were downregulated. Among the upregulated genes in HRS10 after DMI treatment, in ion transport pathways was observed (*P* = 0.0025). In contrast, HRI11 exhibited 17 upregulated genes and 14 downregulated genes post-DMI treatment (**Figure 3**). Notably, gene induced by DMI treatment in HRI11 were enriched in processes related to ergosterol and lipid biosynthesis. The differences following DMI treatment between HRS10 and HRI11 suggest potential mechanisms contributing to resistance in HRI11.

In both isolates, six genes showed elevated expression following propiconazole treatment, including three ergosterol biosynthesis genes (*ERG6*, *ERG11*, and *ERG24*), the ABC transporter *Sh*atrD, a potential histidine kinase/Hsp90-like ATPase, and a serine-type peptidase. Notably, the expression levels of these ergosterol biosynthesis genes were slightly higher in isolate HRI11 compared to isolate HRS10. Additionally, we observed the upregulation of the previously reported ABC transporter *Sh*atrD (Sang et al., 2015) in both isolates after propiconazole treatment. However, another ABC transporter, *Sh*PDR1 (Sang et al., 2018), was induced specifically in the resistant isolate HRI11 in the presence of propiconazole. Similarly, *ERG5* and the previously reported *ShCYP561* were induced exclusively in the resistant isolate following propiconazole treatment. Regarding down-regulated gens, no overlap was found between HRS10 and HRI11. The genes inhibited by DMI in HRI11 were enriched in the mycotoxin biosynthetic process (*P* = 0.002), suggesting an energy allocation strategy that may contribute to resistance mechanisms.

**Figure 4:**
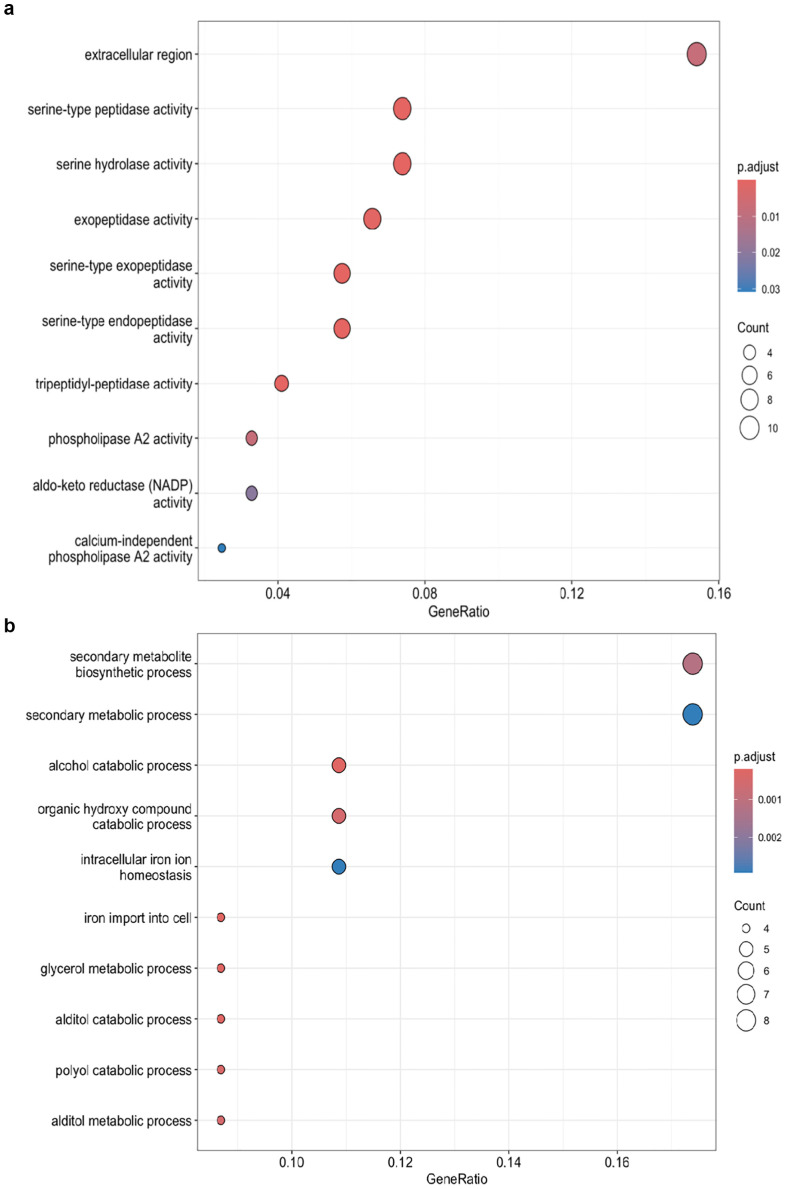
GO enrichment analysis of DEGs in the multidrug-resistant HRI11 isolate compared to the sensitive HRS10 isolate under normal conditions, **(a)** overexpress DEGS and **(b)** repressed DEGs.

Gene expression patterns were compared between two isolates of *C. jacksonii* under normal conditions and after propiconazole treatment to understand their differences. Under normal growth conditions, isolate HRI11 exhibited 245 upregulated genes enriched in extracellular regions, serine peptidase activity, and hydrolase activity (**Figure 4a**), while 110 genes were downregulated and enriched in secondary metabolic processes (**Figure 4b**) compared to the sensitive isolate HRS10. These transcriptomic patterns may reflect shifts in cellular priorities or physiological states between the two isolates. Propiconazole treatment induced additional expression differences between the isolates, with 322 genes upregulated and 146 genes downregulated. GO-enrichment analysis showed similar results compared to non-DMI treatment conditions.

**Figure 5:**
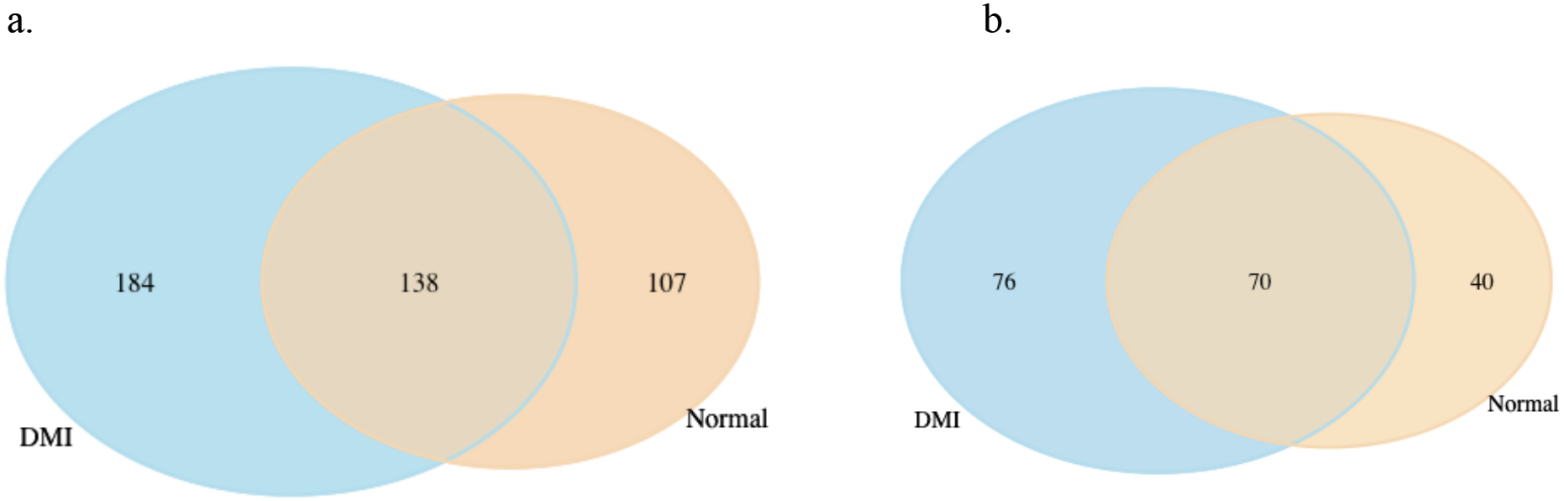
Comparison of differentially expressed genes (DEGs) in HRI11 and HRS10 under normal conditions and DMI treatment. **(a)** Overexpressed DEGs: 138 DEGs are shared between DMI-treated and normal conditions, with 184 unique to DMI-treated and 107 unique to normal conditions. **(b)** Repressed DEGs: 70 DEGs are shared, with 76 unique to DMI-treated and 40 unique to normal conditions.

To identify genes specifically responsive to propiconazole treatment, we further analyzed genes that were uniquely differentially expressed in HRI11 after DMI treatment. After filtering, we identified 184 overexpressed genes and 76 suppressed genes that responded to propiconazole in HRI11 (**Figure 5**). KEGG pathway enrichment analysis revealed that those overexpressed genes were significantly enriched in transporters [Ko02000].

### COG annotation

**Figure 6:**
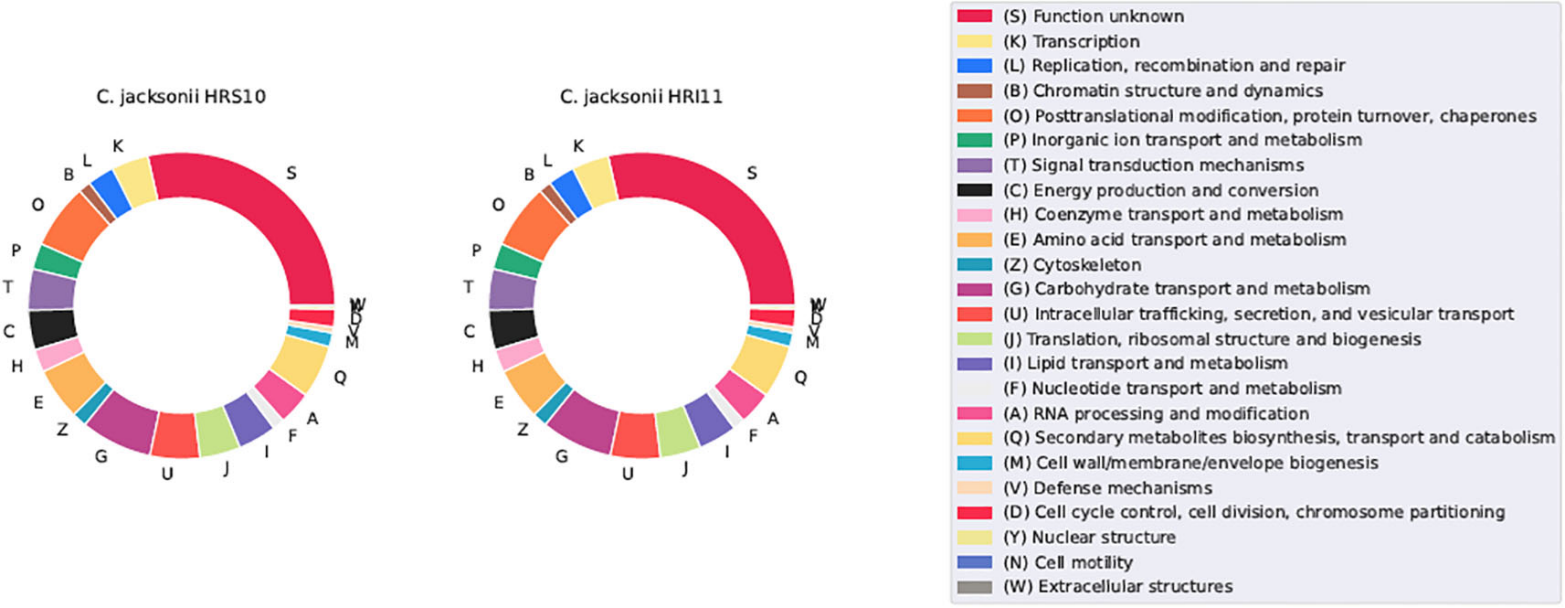
COGs cluster distribution in two *C. jacksonii* isolates: the multi-drug resistant HRI11 and the sensitive HRS10.

The Clusters of Orthologous Groups of Proteins (COGs) database (Galperin et al., 2021) was used to classify protein activities phylogenetically, providing a comprehensive comparison between different isolates. Post-annotation, approximately 29% of protein functions remain unidentified. Known functional families predominantly include: (G) Carbohydrate transport and metabolism (28.5% in HRS10, 28.8% in HRI11), (O) Posttranslational modification, protein turnover, chaperones (7.5% in HRS10, 7.3% in HRI11), (Q) Secondary metabolite biosynthesis, transport (6.8% in HRS10, 6.7% in HRI11), and catabolism, (E) Amino acid transport and metabolism (5.6% in HRS10, 5.9% in HRI11), and (U) Intracellular trafficking, secretion, and vesicular transport (5.2% for both strains) (**Figure 6**). Despite its larger genome size, HRI11 typically possesses either more or the equivalent number of proteins in all COG categories compared to HRS10, except for category (V) Defense mechanisms. HRS10 has 59 proteins in this category, while HRI11 has 58 proteins, a finding that prompts further investigation, as transporters are often involved in fungicide resistance.

Both major facilitator superfamily (MFS) transporters and ATP-binding cassette (ABC) transporters play crucial roles in maintaining cellular homeostasis, influencing various physiological processes such as nutrient uptake, drug resistance, and toxin elimination. Enhanced drug efflux via MFS and ABC transporters has been associated with DMI resistance in diverse fungi (Sirim et al., 2009; Ziogas & Malandrakis, 2015).

HRS10 harbored 353 MFS transporters, whereas HRI11 possessed 375 MFS transporters as identified by InterProScan (Jones et al., 2014), with no SNPs detected among them. The response of MFS transporters to DMI treatment varied between the isolates: in HRS10, two MFS transporters were upregulated and two were downregulated after DMI treatment. In contrast, HRI11 exhibited no significant differential expression of MFS transporters between untreated and DMI-treated conditions, suggesting a limited transcriptional response to the fungicide within this isolate. However, when comparing the two isolates under normal conditions, HRI11 exhibited three upregulated and nine downregulated MFS transporters compared to HRS10, indicating a potentially pre-existing resistance mechanism in HRI11 influencing MFS transporter expression. Post-DMI treatment, HRI11 showed 13 overexpressed and 12 inhibited MFS transporters. The greater number of MFS transporters and their diverse expression patterns in HRI11, particularly under DMI treatment, underscore potential regulatory mechanisms driving differential responses in the two *C. jacksonii* isolates.

In addition to MFS transporters, HRS10 and HRI11 possessed 65 and 77 ABC transporters, respectively. Two non-synonymous SNPs were identified in two ABC transporters, including *ShatrD*. Beyond genetic variation, the expression of ABC transporters varied across different conditions. *ShatrD* was upregulated in response to DMI treatment in both HRS10 and HRI11. Moreover, *ShatrD* showed higher expression in HRI11 than in HRS10 under both untreated and treated conditions, highlighting its pivotal role in responding to DMI and in resistance mechanisms. *ShPDR1* exhibited upregulation, specifically in HRI11 following DMI treatment and maintained higher expression levels in HRI11 compared to HRS10 across all conditions, indicating its potential involvement in baseline resistance in HRI11. Additionally, *FUN_007147-T1* (hypothetical protein) showed elevated expression in HRI11 compared to HRS10 regardless of DMI treatment, suggesting its potential involvement in the inherent resistance mechanisms of HRI11. Moreover, *MDR1_1* and *MDR1_3* were specifically upregulated in HRI11 under DMI treatment.

### CAZyme annotation

The Carbohydrate-Active enZYme (CAZy) database complies the enzymes involved in the synthesis, metabolism, and reorganization of complex carbohydrates. Predictions identified 512 and 528 CAZyme-encoding genes in HRS10 and HRI11, respectively (**Supplementary Figure S1**). The putative CAZymes were assigned into six classes: Auxiliary Activities (AA), Carbohydrate-Binding Modules (CBM), Carbohydrate Esterases (CE), Glycoside Hydrolases (GH), Glycosyl Transferases (GT), and Polysaccharide Lyases (PL). Compared with HRS10, families of GT maintained the same and families of CBM contracted while the other families expanded in HRI11.

### Transcriptional factors

Transcription factors (TFs) play pivotal roles in diverse biological processes such as development, metabolism, stress response, and pathogenicity including drug resistance in filamentous fungi (Cacciotti et al., 2024; John et al., 2021, 2022). In HRS10 and HRI11, a total of 367 and 387 TFs were predicted, respectively. More than half of these TFs contain the fungal Zn_2_-Cys_6_ binuclear cluster domain and/or the fungal-specific TF domain, crucial for regulating drug efflux pump activity (MacPherson et al., 2006). A previous study (Sang et al., 2018) highlighted that HRI11 features an amino acid substitution in the Zn_2_-Cys_6_ TF *ShXDR1*, contributing to multidrug resistance by upregulating efflux pump encoding genes such as *ShatrD* and *ShPDR1*. Additionally, HRI11 possesses three unique TFs: one MADS-box superfamily TF, one CCHC-type zinc finger TF, and one TF with a Zn_2_-Cys_6_ fungal-type DNA-binding domain.

**Figure 7:**
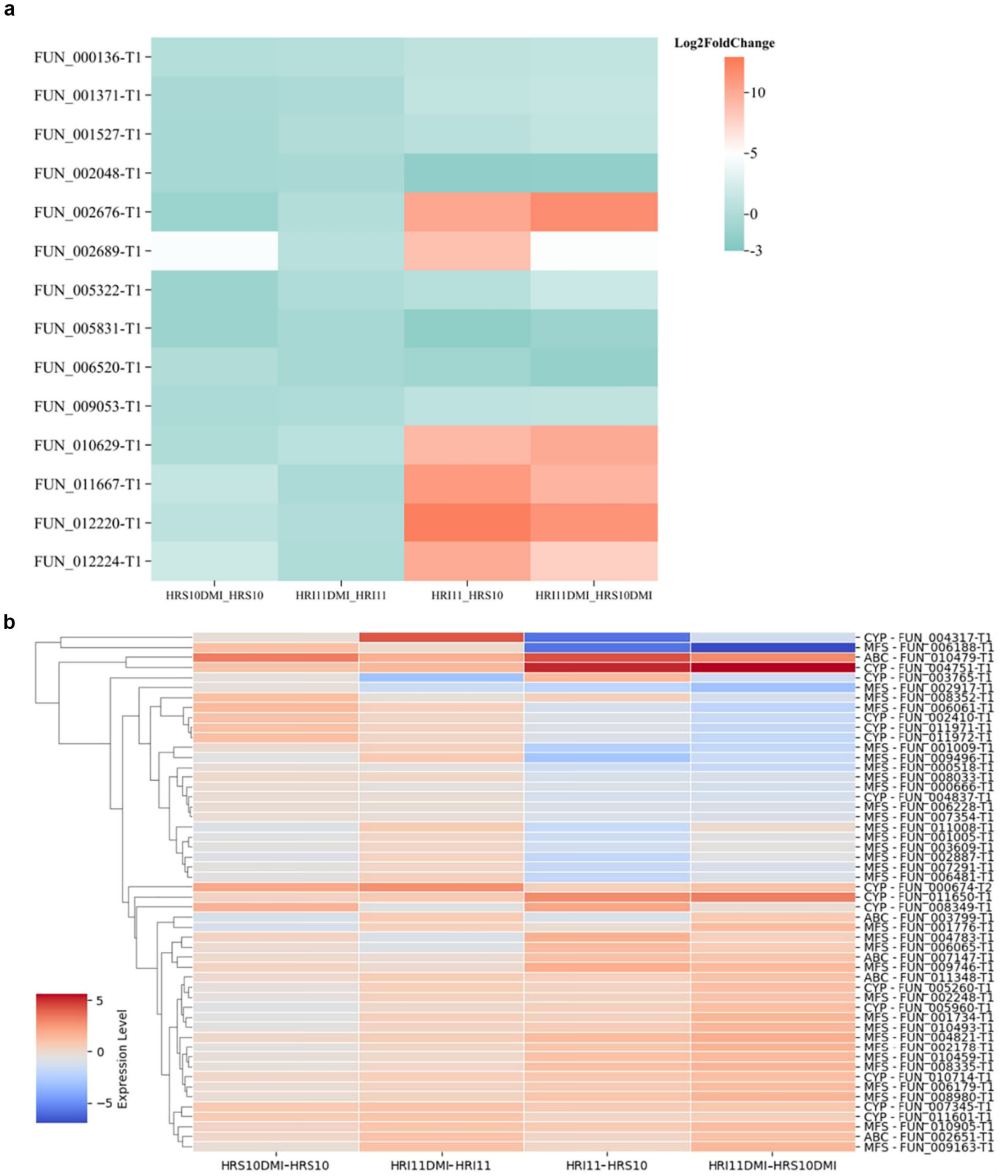
Gene expression of drug resistance-related gene families under various conditions in two *C. jacksonii* isolates: the multi-drug resistant HRI11 and the sensitive HRS10. **(a)** transcription factors, and **(b)** ABC, MFS and CYP families. Comparison include: HRS10DMI vs. HRS10: HRS10 under DMI treatment vs. untreated; HRI11DMI vs. HRI11: HRI11 under DMI treatment vs. untreated; HRI11 vs. HRS10: HRI11 untreated vs. HRS10 untreated; HRI11DMI vs. HRS10DMI: HRI11 under DMI treatment vs. HRS10 under DMI treatment. **(a)** Transcription factors. **(b)** ABC, MFS and CYP families.

The expression level of TFs under different conditions were compared (**Figure 7a**), revealing distinct regulatory patterns in HRI11 following exposure to DMI fungicide. After propiconazole treatment, HRI11 showed no significant change in TFs expression, whereas HRS10 exhibited downregulation of two TFs. Under normal conditions compared to HRS10, HRI11 had four TFs upregulated and two TFs downregulated. Following DMI treatment, HRI11 displayed upregulation of 10 TFs and inhibition of three TFs. These findings highlight a differential regulatory system in HRI11, particularly in response to fungicide stress, potentially influencing its adaptive capabilities and drug resistance mechanisms.

### Gene families with vital roles in drug resistance are conserved in both isolates

**Figure 8:**
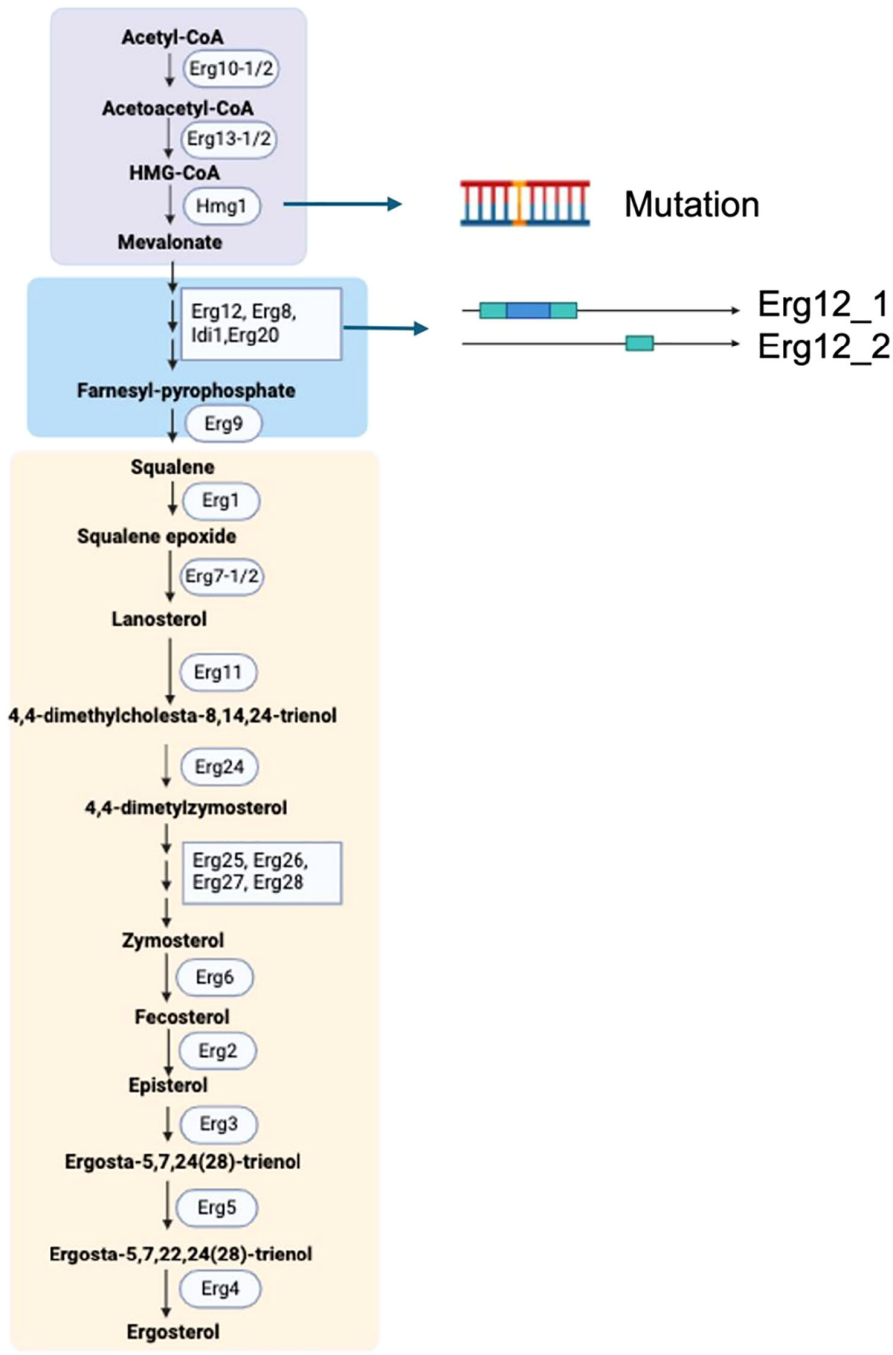
Ergosterol biosynthetic pathway in *C. jacksonii.* The pathway is divided into three modules: the mevalonate pathway (violet box), farnesyl pyrophosphate biosynthesis (blue box), and the late ergosterol pathway (peach-colored box). Key genomic variations in the resistant isolate HRI11, compared to the sensitive isolate HRS10, include a point mutation in *Hmg1* and the presence of Erg12 paralogs. The enzymes involved in this pathway are as follows: Erg10, acetyl-CoA C-acetyltransferase; Erg13, HMG-CoA synthase; Hmg1, HMG-CoA reductase; Erg12, mevalonate kinase; Erg8, phosphomevalonate kinase; Idi1, isopentenyl diphosphate isomerase; Erg20, farnesyl pyrophosphate synthetase; Erg9, squalene synthase; Erg1, squalene epoxidase; Erg7, lanosterol synthase; Erg11 (Cyp51), lanosterol C-14 demethylase; Erg24, sterol C-14 reductase; Erg25, sterol C-4 methyloxidase; Erg26, sterol C-3 dehydrogenase (C4-decarboxylase); Erg27, sterol C-3 ketoreductase; Erg6, sterol C-24 methyltransferase; Erg2, sterol C-8 isomerase; Erg3, sterol C-5 desaturase; Erg5, sterol C-22 desaturase; Erg4, sterol C-24 reductase; and Erg28, ergosterol-associated protein. Created in https://BioRender.com.

Ergosterol, the primary fungal sterol on the cell membranes, significantly influences membrane integrity, permeability, and fluidity (Rodrigues, 2018). It is also a target for many antifungal medicines (Gulshan & Moye-Rowley, 2007). We investigated ergosterol biosynthesis genes at different levels to understand their role in drug resistance. Both isolates share 26 identical ergosterol synthesis genes (**Figure 8**). HRI11 possesses an additional *ERG12_2* paralog and a non-synonymous substitution in *HMG1*. The effects of propiconazole on three ergosterol biosynthesis genes *ERG6*, *ERG11*, and *ERG24* were evaluated in both isolates.

Additionally, cytochrome P450 monooxygenases (CYPs) are widely recognized for their role in detoxifying xenobiotics and contributing to fungicide resistance (Lah et al., 2011). HRS10 and HRI11 harbor 106 and 126 CYPs, respectively. Apart from the higher count of CYPs in HRI11, it also exhibits a non-synonymous SNPs in FUN_010664 compared to HRS10. Previous studies have shown that *ShCYP65* is upregulated in HRI11 compared to HRS10 under normal conditions and after DMI treatment, implicating its potential involvement in the fungicide resistance mechanism of HRI11. Similarly, *ShCYP561* responds similarly to *ShCYP65*, showing upregulation after DMI treatment in HRI11. Several other CYPs, including *FUN_000674-T2*, are implicated in DMI resistance, with elevated expression levels following DMI treatments in both isolates and higher expression in HRI11 compared to HRS10 post-DMI treatment. In HRS10, six CYPs were up-regulated and three were down-regulated after propiconazole treatment, whereas HRI11 exhibited upregulation of seven genes and downregulation of two CYP genes. Compared to HRS10, *FUN_010440* in HRI11 was repressed under normal conditions but overexpressed after propiconazole treatment. However, *FUN_008349-T1* is not differentially expressed to DMI in HRI11 (**Figure 7b**).

Glutathione S-transferases (GSTs) constitute a diverse family of enzymes crucial for detoxifying various endogenous and xenobiotic compounds, including fungicides. These enzymes facilitate detoxification by catalyzing the conjugation of the tripeptide glutathione to a wide array of substrates, thereby neutralizing their toxicity and promoting their elimination from the cell (Sheehan et al., 2001). Both isolates were found to possess 26 GSTs. Among them, *FUN_005805-T1* shows upregulation in HRI11 compared to HRS10 under normal conditions, while *FUN_010740-T1* exhibits upregulation in HRI11 after DMI treatment. This differential expression suggests a possible role for these GSTs in augmenting detoxification capacity and potentially contributing fungicide resistance in HRI11.

Additionally, we examined 39 known genes, including *Yap1*, *Ecm1*, *etc.* which are implicated in drug resistance. However, no differences in expression were observed between the two isolates in terms of these genes.

### The resistome in *Clarireedia jacksonii*

Using a sequence similarity threshold of 60%, we identified 23 resistance genes that showed no differences between the two isolates, including the DMI target gene *ERG11*/*CYP51*. However, the *beta-tubulin 2* gene, which is the target of methyl benzimidazole carbamate fungicides, exhibited an E198A amino acid substitution in the MDR isolate HRI11 compared to the sensitive isolate HRS10. This mutation, known to confer benzimidazole resistance, has been widely documented in other pathogenic fungi such as *Venturia inaequalis, Gibberella zeae*, and various C. *jacksonii* isolates.

## Discussion

Despite the economic importance of dollar spot disease on turfgrass, no high-quality genome assemblies have been available for its causal agents, *Clarireedia* spp. To date, available genomic resources are limited to fragemented draft assemblies of a few *Clarireedia jacksonii* and *C. monteithiana* isolates, many of which vary significantly in size and scaffold number (Sapkota et al., 2022). This lack of comprehensive genomic data has hindered in-depth investigations into pathogenicity, fungicide resistance, and species divergence within the genus. Our study addresses this critical gap by presenting the most complete and contiguous genome assemblies to date for *C. jacksonii*, generated from a comparative analysis of a highly DMI-resistant isolate (HRI11) and a fungicide-sensitive isolate (HRS10). The isolates HRS10 and HRI11 exhibit significant differences in fungicide sensitivity, with HRI11 demonstrating resistance across multiple fungicide classes and over a 40-fold reduction in sensitivity to propiconazole compared to the sensitive isolate HRS10 (Hulvey et al., 2012). These contrasting phenotypes provide an ideal system for investigating the genetic basis of DMI fungicide resistance in *Clarireedia* spp.

To achieve high-quality genome assemblies, we employed a hybrid genome assembly approach integrating Oxford Nanopore, PacBio, and Illumina sequencing technologies. This strategy substantially improved the assemblies for both isolates, reducing scaffold numbers to one-fourth and one-half of those reported in earlier version (Green et al., 2016). Genome completeness, assessed using BUSCO, further confirmed the quality of these assemblies, with 98% of expected single-copy orthologs identified in both isolates. Beyond improving scaffold continuity, Nanopore sequencing enabled the effective characterization of repetitive DNA and structural variations (Sedlazeck et al., 2018), resulting in a 6% reduction in genome size for HRS10 compared to previous versions. Repeative elements are a common source of misassemblies, fragmentation, and artificial genome inflation, often resulting in inaccurate gene predictions and unreliable structural variant detection (Alkan et al., 2011). Long-read sequencing addresses this challenge by spanning complex, repeat-rich regions, thereby improving assembly contiguity and enabling more accurate, repeat annotation and filtering (van Dijk et al., 2018). Notably, the genome of HRI11 is approximately 10% larger than that of HRS10, likely due to an increase in repetitive sequences. These improved genome resources establish a robust foundation for investigations into genome structure, gene function, and fungicide resistance mechanisms in this economically significant turfgrass pathogen.

The genomic analysis of the resistant isolate HRI11 in comparison to the sensitive isolate HRS10 revealed significant SVs, CNVs, and SNPs that likely contribute to the resistance phenotype observed in HRI11. Insertion events were particularly impactful, introducing frameshifts in the multidrug resistance gene *MDR1_3*, which may alter its functionality and contribute directly to fungicide resistance. Additionally, a breakpoint in *NUD1*, encoding a leucine-rich repeat (LRR) protein, in HRI11 suggests a potential disruption of this gene’s function. LRR proteins are known to mediate protein-protein interactions and play in roles in signaling pathways related to stress responses and pathogen defense in both fungi and plants (Kobe and Kajava, 2001; Ng and Xavier, 2011). Disruption of *NUD1* may therefore alter cellular signaling under fungicide-induced stress, potentially contributing to the resistance phenotype observed in HRI1. Deletions of *FSH3* (serine hydrolase) and FUN_011878, combined with the duplication of FUN_011921, further illustrate how SVs can generate genomic diversity that impacts stress-related pathways and resistance mechanisms. In *Saccharomyces cerevisiae*, FSH3 is upregulated in response to oxidative stress and promotes cell death through apoptotic pathways. Deletion of FSH3 enhances cell survival under such conditions, whereas its overexpression leads to growth inhibition (Gowsalya et al., 2019). The absence of FSH3 in the resistant isolate HRI11 may indicate a comparable adaptation, reflecting a shift in stress response strategy that favor fungal survival under fungicide-induced oxidative pressure.

CNVs in HRI11 revealed an increased CN in 31 genes and decreased CN in 29 genes. Notably, *SGE1*, a global transcription regulator, exhibited increased CN alongside an impactful SNP. The dual alteration of *SGE1* is particularly significant, as it may amplify transcriptional activity and introduce functional modifications, that enhance the expression of downstream genes under fungicide stress. SGE1 belongs to the highly conserved Wor1-like (WOPR) family of regulators known to coordinate virulence and stress response pathways in numerous plant pathogenic fungi. For example, in *Fusarium oxysporum*, *SGE1* is essential for the activation of SIX effector genes and is required for full virulence (Michielse et al., 2009). Similarly, studies in *F. verticillioides* and *F. graminearum* have shown that *SGE1* or its homologs regulate large networks of effectors, secondary metabolite genes, and stress-response genes, and are also responsive to environmental cues such as oxidative and osmotic stress (Brown et al., 2014; Jonkers et al., 2012). Therefore, the combined CNV and SNP observed here may enhance the ability of SGE1 to mediate transcriptional responses to fungicide pressure, contributing to resistance through coordinated activation of detoxification, efflux, or membrane repair pathways. These findings position *SGE1* as a compelling candidate for functional validation and as a potential marker or target in the broader context of fungicide resistance mechanisms.

Over half of the genes with increased copy number in HRI11 are tRNA genes, including tRNA-Lys, tRNA-Gly, and tRNA-ArgClick or tap here to enter text., suggesting a potential link between increased tRNA gene copy number and codon usage bias. In highly expressed genes, codon usage tends to favor ‘optimal’ codons, whose correspondto more abundant tRNAs, thereby enhancing translational efficiency (dos Reis et al., 2004; Gingold and Pilpel, 2011; Sharp and Li, 1986). More importantly, in *S.cerevisiae*, increased tRNA gene copy number has been linked to enhanced translational efficiency under stress conditions (Yona et al., 2013). In HR111, many of the expanded tRNA genes are clustered within scaffold 38, often in gene-dense regions or putative genomic islands, suggesting localized genome plasticity. This plasticity may result from segmental duplications or transposon activity-mechanisms known to drive genome adaptation in filamentous fungi (Faino et al., 2015; Möller and Stukenbrock, 2017).

Therefore, although it remains unclear whether the increased tRNA gene copy number directly contributes to DMI resistance, its enrichment in genomic clusters—especially in a stress-adapted isolate like HRI11—raises the possibility that it supports more efficient translation of resistance-associated proteins. These expansions may represent adaptive responses to fungicide pressure or, alternately, may be by products of broader genome remodeling processes that accompany resistance evolution. To clarify the functional significance of tRNA gene expansion, future studies should integrate codon usage analysis, transcriptomic profiling, and ribosome occupancy data (e.g., Ribo-seq) to assess its impact on translational efficacy and fungicide response.

Among the 12 copy number increased mRNA genes are *nad5* (NADH: ubiquinone oxidoreductase subunit 5), which supports mitochondrial function under stress, and 10 hypothetical proteins with unknown but potentially critical roles. Conversely, decreased CNs were observed exclusively in mRNA genes, including the ATP-dependent RNA helicases *DED1* and *CHL1*, potentially deprioritizing RNA processing pathways in favor of stress-response mechanisms.

A comprehensive annotation process identified over a thousand putative genes across several databases. Due to their close genetic relationship, HRS10 and HRI11 share the majority of their genome content and functional proteins, despite HRI11 having a larger genome size. A result, HRI11 harbors more protein-coding genes across most functional categories compared to HRS10, including gene families associated with drug resistance and transcription factors. Carbohydrate and energy metabolism are fundamental processes that enable organisms to function and respond to environment changes (Kan et al., 2019). The differences of CAZyme-encoding genes suggest that HRI11 may possess enhanced capabilities in key biological processes such as polysaccharide degradation and glycoside hydrolases, potentially contributing to its heightened resistance. Additional genes in transporters and metabolism-related families likely equip HRI11 with improved adaptive responses to fungicide exposure.

Additionally, RNA-Seq transcriptome analysis was conducted to investigate differential transcriptional responses to propiconazole treatment between the HRS10 and HRI11 isolates. To prioritize the identification of resistance-associated genes and pathways, all RNA-seq reads were aligned to the HRI11 reference genome. While this approach enables consistent comparisons within a resistance-focused genomic framework, it introduces an important limitation: gene expression in the sensitive isolate HRS10 is only assessed for genes that are present, conserved, and mappable within the HRI11 genome. Consequently, genes unique to HRS10 or those with substantial sequence divergence may be underrepresented or excluded from downstream analysis. Nevertheless, aligning all reads to the HRI11 genome enables consistent, resistance-focused comparisons of gene expression across isolates, emphasizing mechanisms embedded within the resistant genomic background.

Despite this constraint, the two isolates exhibited distinct transcriptional responses to DMI exposure. In HRS10, up-regulated genes were enriched in ion transport functions, whereas in HRI11, enrichment was observed in ergosterol and lipid biosynthesis pathways-classic targets of azole antifungal activity. Genes uniquely up-regulated in HRI11 included those associated with the extracellular region, serine peptidase activity, and serine hydrolase activity (**Figure 4a**), suggesting a potential role for enhanced extracellular interactions and enzymatic detoxification in resistance. Meanwhile, down-regulated genes in HRI11 were enriched in secondary metabolic processes (**Figure 4b**), suggesting a potential reallocation of metabolic resources away from secondary metabolism toward more immediate stress responses. However, these interpretations should be considered preliminary due to inherent limitations of the RNA-seq approach, particularly the reliance on a single reference genome. Nevertheless, the observed expression patterns in HRI11-including elevated levels of transporters, cytochrome P450s, and transcription factors-align with established resistance-associated pathways and warrant further experimental validation.

Interestingly, although propiconazole is known to inhibit fungal growth by blocking ergosterol biosynthesis and downregulating associated gene expression, both isolates showed increased expression of three ergosterol biosynthetic genes shortly after treatment. This response likely reflects the rapid activation of fungal xenobiotic detoxification mechanisms, occurring within one hour of fungicide exposure-before irreversible cellular damage could occur-resulting in enhanced production of ergosterol-related enzymes. A similar early response to azole fungicides has been observed in *Neurospora crassa* (Hu et al., 2018). However, additional time-course experiments would be necessary to fully characterize the dynamics and specificity of this response.

Unlike comprehensive bacterial resistome databases like CARD, fungal resistance databases are limited. For example, AFRbase includes only 22 genes, mainly from *Candida* and *Aspergillus*, while MARDy supports only single-gene queries across 27 fungal species, severly constraining genome-wide resistome analyses. We performed a comprehensive genome-wide resistome analysis by manually retrieving gene sequences from NCBI and utilizing BLAST to investigate the resistome of C. *jacksonii*. This analysis revealed an E198A amino acid substitution in the β-tubulin gene of the multidrug-resistant isolate HRI11. The β-tubulin gene plays a crucial role in fungicide resistance in many plant pathogenic fungi (Chen et al., 2009; Zhu et al., 2010), particularly those resistant to methyl benzimidazole carbamate (MBC) fungicides. Specific mutations in this gene, especially at codons 198 and 200, are strongly associated with resistance to benzimidazole and zoxamide fungicides (Vela-Corcía et al., 2018). This finding underscores the effectiveness of resistome detection methodologies. Therefore, we advocate developing a comprehensive fungal resistome database to rapidly and accurately identify fungicide resistance, integrating bioinformatics with experimental approaches to support effective global fungal disease management.

Next-Generation sequencing has revolutionized the rapid generation of vast DNA sequence data across diverse organisms, significantly advancing genome annotation (Ejigu and Jung, 2020). Historically, genome annotation faced challenges, with about half of newly sequenced genomes two decades ago containing hypothetical proteins of unknown function (Stein, 2001). Despite recent bioinformatics advancements, eukaryotic genome annotation remains complex, with 29% of proteins in our annotation still functionally unknown. This limits our understanding of the relationship between observed gene numbers or expression differences and drug resistance, particularly genes annotated as hypothetical proteins or containing SNPs. Moreover, BLAST searches of *Clarireedia* genes using the NCBI database frequently returns top hits from unrelated fungal species such as *Botrytis* or *Sclerotinia* species, rather than *Clarireedia* spp., even when the query sequence originates from *Clarireedia* itself. This underscores the current lack of well-annotated and complete genomic references for the genus, further emphasizing the need for high-quality genome assemblies. Despite these limitations, our integration of genome and transcriptome data has revealed that resistance in the HRI11 isolate operates through a dual mechanism: (1) baseline resistance conferred by genetic variations in fungicide target genes, transcription factors, and transporters, and (2) inducible resistance through the upregulation of efflux-related and stress-responsive genes upon DMI exposure. The combination of fixed genetic changes and dynamic transcriptional responses underscores the evolutionary flexibility of *C. jacksonii* in adapting to fungicide pressure.

While this comparative study provides valuable insights into the genomic and transcriptional underpinnings of fungicide resistance, we acknowledge the limitation of using only two isolates. Resistance is likely polygenic and shaped by broader population-level variation. Therefore, further studies involving a wider panel of resistant and sensitive isolates will be essential to validate the candidate genes and resistance mechanisms identified here. Functional assays and population-scale genomics will also be critical to confirm the roles of candidate genes affected by copy number variations or differential expression. Nonetheless, this work establishes the first high-quality genomic and transcriptomic framework for *C. jacksonii*, offering a critical foundation for future investigations into the molecular evolution of fungicide resistance in this economically important turfgrass pathogen.

## Data Availability

The datasets presented in this study can be found in online repositories. The names of the repository/repositories and accession number(s) can be found below: https://www.ncbi.nlm.nih.gov/, PRJNA1216260.
