# Supplementary material for: Nanopore sequencing and hybrid assembly: unraveling the genomic landscape of dollar spot with enhanced annotation and drug resistance profiling
Source: Front Fungal Biol. 2025 Jul 17;6:1621663. doi: 10.3389/ffunb.2025.1621663 (PMC12310695; doi:10.3389/ffunb.2025.1621663)
Supplement: Supplementary file 1 [file SupplementaryFile1.docx]

**Supplementary material:**

Fig. S1. CAZyme-encoding genes in HRS10 and HRI11

Table S1. Orthogroups alignment statistics

|  | HRI11 | HRS10 |
| --- | --- | --- |
| Number of protein-coding genes | 12,832 | 12,161 |
| Number of genes in orthogroups | 12,179 | 11,519 |
| Number of unassigned genes | 653 | 643 |
| Percentage of genes in orthogroups | 94.9% | 94.7% |
| Percentage of unassigned genes | 5.1% | 5.3% |
| Number of species-specific orthogroups | 46 | 8 |
| Number of genes in species-specific orthogroups | 145 | 17 |
| Percentage of genes in species-specific orthogroups | 1.1% | 0.1% |

Table S2. Copy number variation gene annotation

S2a. Table of increased copy number variation annotation

| Gene_id | Type | Scaffold | Start | End | Copy numbers | Annotation |
| --- | --- | --- | --- | --- | --- | --- |
| ACMFMF_011951-T1 | mRNA | 4 | 1317689 | 1321657 | 6 | hypothetical protein |
| ACMFMF_011921-T1 | mRNA | 13 | 1072456 | 1075487 | 5 | hypothetical protein |
| ACMFMF_007295-T1 | mRNA | 14 | 36974 | 37330 | 3 | hypothetical protein |
| ACMFMF_011596-T1 | mRNA | 38 | 54444 | 54800 | 20 | hypothetical protein |
| ACMFMF_011600-T1 | mRNA | 38 | 86042 | 87138 | 20 | NADH:ubiquinone oxidoreductase subunit 5 |
| ACMFMF_011602-T1 | mRNA | 38 | 96324 | 96629 | 24 | hypothetical protein |
| ACMFMF_011606-T1 | mRNA | 38 | 115994 | 116329 | 24 | hypothetical protein |
| ACMFMF_011607-T1 | mRNA | 38 | 120364 | 121668 | 24 | hypothetical protein |
| ACMFMF_011613-T1 | mRNA | 38 | 174340 | 174684 | 24 | hypothetical protein |
| ACMFMF_011595-T1 | tRNA | 38 | 23016 | 23087 | 20 | tRNA-Lys |
| ACMFMF_011597-T1 | tRNA | 38 | 67476 | 67547 | 20 | tRNA-Lys |
| ACMFMF_011598-T1 | tRNA | 38 | 67829 | 67899 | 20 | tRNA-Gly |
| ACMFMF_011599-T1 | tRNA | 38 | 69709 | 69779 | 20 | tRNA-Arg |
| ACMFMF_011601-T1 | tRNA | 38 | 87305 | 87376 | 20 | tRNA-Val |
| ACMFMF_011603-T1 | tRNA | 38 | 108607 | 108678 | 24 | tRNA-Ile |
| ACMFMF_011604-T1 | tRNA | 38 | 113057 | 113128 | 24 | tRNA-Ile |
| ACMFMF_011605-T1 | tRNA | 38 | 113315 | 113400 | 24 | tRNA-Ser |
| ACMFMF_011608-T1 | tRNA | 38 | 123840 | 123912 | 24 | tRNA-Glu |
| ACMFMF_011609-T1 | tRNA | 38 | 124646 | 124718 | 24 | tRNA-Met |
| ACMFMF_011610-T1 | tRNA | 38 | 124741 | 124822 | 24 | tRNA-Leu |
| ACMFMF_011611-T1 | tRNA | 38 | 126853 | 126925 | 24 | tRNA-Phe |
| ACMFMF_011612-T1 | tRNA | 38 | 132031 | 132103 | 24 | tRNA-Met |
| ACMFMF_011614-T1 | tRNA | 38 | 179505 | 179575 | 24 | tRNA-Arg |
| ACMFMF_011615-T1 | tRNA | 38 | 183147 | 183217 | 24 | tRNA-Lys |
| ACMFMF_011616-T1 | tRNA | 38 | 199732 | 199803 | 24 | tRNA-Lys |
| ACMFMF_011617-T1 | tRNA | 38 | 199884 | 199954 | 24 | tRNA-Gly |
| ACMFMF_011618-T1 | tRNA | 38 | 207477 | 207547 | 24 | tRNA-Asn |
| ACMFMF_011619-T1 | tRNA | 38 | 207569 | 207640 | 24 | tRNA-Gln |
| ACMFMF_011877-T1 | mRNA | 57 | 3588 | 4625 | 6 | Global transcription regulator sge1 |
| ACMFMF_011878-T1 | mRNA | 57 | 10765 | 12724 | 6 | hypothetical protein |
| ACMFMF_011879-T1 | mRNA | 57 | 19032 | 20498 | 6 | hypothetical protein |

S2b. Table of decreased copy number variation annotation

| Gene_id | Scaffold | Start | End | Annotation |
| --- | --- | --- | --- | --- |
| ACMFMF_000981-T1 | 1 | 3121570 | 3123504 | hypothetical protein |
| ACMFMF_011951-T1 | 4 | 1317689 | 1321657 | hypothetical protein |
| ACMFMF_004403-T1 | 7 | 547052 | 548892 | hypothetical protein |
| ACMFMF_004599-T1 | 7 | 1147991 | 1148593 | hypothetical protein |
| ACMFMF_004600-T1 | 7 | 1149310 | 1150723 | hypothetical protein |
| ACMFMF_005419-T1 | 9 | 691344 | 692578 | hypothetical protein |
| ACMFMF_005420-T1 | 9 | 693032 | 694483 | hypothetical protein |
| ACMFMF_005421-T1 | 9 | 694499 | 699027 | DEAD-box ATP-dependent RNA helicase |
| ACMFMF_005642-T1 | 9 | 1466804 | 1468125 | hypothetical protein |
| ACMFMF_005643-T1 | 9 | 1468713 | 1470806 | hypothetical protein |
| ACMFMF_005644-T1 | 9 | 1472619 | 1474268 | hypothetical protein |
| ACMFMF_005645-T1 | 9 | 1475023 | 1476175 | hypothetical protein |
| ACMFMF_007466-T1 | 14 | 574624 | 577302 | ATP-dependent DNA helicase chl1 |
| ACMFMF_007467-T1 | 14 | 577650 | 579839 | hypothetical protein |
| ACMFMF_011934-T1 | 24 | 59331 | 61101 | hypothetical protein |
| ACMFMF_011363-T1 | 35 | 10985 | 12022 | hypothetical protein |
| ACMFMF_011364-T1 | 35 | 12828 | 15118 | hypothetical protein |
| ACMFMF_011797-T1 | 48 | 17935 | 19859 | hypothetical protein |
| ACMFMF_011798-T1 | 48 | 20030 | 21144 | hypothetical protein |
| ACMFMF_011799-T1 | 48 | 21335 | 21755 | hypothetical protein |
| ACMFMF_011800-T1 | 48 | 22502 | 23941 | hypothetical protein |
| ACMFMF_011801-T1 | 48 | 27456 | 28393 | hypothetical protein |
| ACMFMF_011802-T1 | 48 | 29114 | 29574 | hypothetical protein |
| ACMFMF_011814-T1 | 48 | 70667 | 72153 | hypothetical protein |
| ACMFMF_011851-T1 | 52 | 11584 | 11964 | hypothetical protein |
| ACMFMF_011852-T1 | 52 | 21559 | 21939 | hypothetical protein |
| ACMFMF_011853-T1 | 52 | 31544 | 31924 | hypothetical protein |
| ACMFMF_011854-T1 | 52 | 41519 | 41899 | hypothetical protein |
| ACMFMF_011892-T1 | 73 | 1494 | 4199 | hypothetical protein |
